# Complement system activation contributes to the ependymal damage induced by microbial neuraminidase

**DOI:** 10.1186/s12974-016-0576-9

**Published:** 2016-05-21

**Authors:** Pablo Granados-Durán, María Dolores López-Ávalos, Timothy R. Hughes, Krista Johnson, B. Paul Morgan, Paul P. Tamburini, Pedro Fernández-Llebrez, Jesús M. Grondona

**Affiliations:** Departamento de Biología Celular, Genética y Fisiología, IBIMA, Facultad de Ciencias, Universidad de Málaga, Málaga, 29071 Spain; Institute of Infection and Immunity, School of Medicine, Cardiff University, Cardiff, UK; Alexion Pharmaceuticals Inc., 352 Knotter Drive, Cheshire, CT 06410 USA

**Keywords:** Complement system, Neuraminidase, Ependymal cells, Inflammation, Brain ventricles, Anti-C5, C6-deficient rats

## Abstract

**Background:**

In the rat brain, a single intracerebroventricular injection of neuraminidase from *Clostridium perfringens* induces ependymal detachment and death. This injury occurs before the infiltration of inflammatory blood cells; some reports implicate the complement system as a cause of these injuries. Here, we set out to test the role of complement.

**Methods:**

The assembly of the complement membrane attack complex on the ependymal epithelium of rats injected with neuraminidase was analyzed by immunohistochemistry. Complement activation, triggered by neuraminidase, and the participation of different activation pathways were analyzed by Western blot. In vitro studies used primary cultures of ependymal cells and explants of the septal ventricular wall. In these models, ependymal cells were exposed to neuraminidase in the presence or absence of complement, and their viability was assessed by observing beating of cilia or by trypan blue staining. The role of complement in ependymal damage induced by neuraminidase was analyzed in vivo in two rat models of complement blockade: systemic inhibition of C5 by using a function blocking antibody and testing in C6-deficient rats.

**Results:**

The complement membrane attack complex immunolocalized on the ependymal surface in rats injected intracerebroventricularly with neuraminidase. C3 activation fragments were found in serum and cerebrospinal fluid of rats treated with neuraminidase, suggesting that neuraminidase itself activates complement. In ventricular wall explants and isolated ependymal cells, treatment with neuraminidase alone induced ependymal cell death; however, the addition of complement caused increased cell death and disorganization of the ependymal epithelium. In rats treated with anti-C5 and in C6-deficient rats, intracerebroventricular injection of neuraminidase provoked reduced ependymal alterations compared to non-treated or control rats. Immunohistochemistry confirmed the absence of membrane attack complex on the ependymal surfaces of neuraminidase-exposed rats treated with anti-C5 or deficient in C6.

**Conclusions:**

These results demonstrate that the complement system contributes to ependymal damage and death caused by neuraminidase. However, neuraminidase alone can induce moderate ependymal damage without the aid of complement.

## Background

The ependyma is a multiciliated cubic epithelium that covers the ventricular cavities of the central nervous system (CNS) in chordates. It contributes to the cerebrospinal fluid (CSF) flow through cilia beating, and serves as a morphological and functional barrier between the CSF and the CNS parenchyma, as it controls the movement of molecules from one compartment to the other. Thus, the ependyma regulates CSF composition [1–3]. The ependymal cells are considered part of the subventricular zone niche located in the lateral ventricles in the adult brain [4] and contribute to neurogenesis by producing regulatory factors [5]. Some authors have even proposed that ependymal cells themselves could act as neural stem cells in adults [6].

Therefore, the loss of the ependymal layer represents a serious, and irreparable, injury to the CNS with consequences for CSF homeostasis [7, 8]. Furthermore, the absence of the ependymal barrier facilitates the access of pathogens and inflammatory cells from the CSF to the parenchyma. This could be related, in the long run, to the development of demyelinating or neurodegenerative disorders [9].

A relationship has been established between CSF infection by viruses, particularly myxoviruses (mumps, influenza, and measles), and ependymal death. The latter has also been linked to the development of hydrocephalus [7, 10–13]. An association has also been reported between viral infections during pregnancy or infancy and subsequent degenerative pathologies in the adult. Such is the case for the Epstein-Barr virus (which causes infectious mononucleosis) and multiple sclerosis [14, 15], influenza virus infection during pregnancy and schizophrenia in the adult offspring [16, 17], and measles infection and subacute sclerosing panencephalitis [18, 19]. In most cases, it is the inflammatory process following the infection that directly causes the neurodegeneration [20]. The fact that both ependymal loss and neurodegenerative disorders had been associated with the same infectious agents indicates that a relationship between these processes could exist. It is conceivable that the ependymal loss caused by infections might allow access to CNS parenchyma of the infectious agent and the inflammatory cells, these being responsible for the subsequent neurodegeneration [9]. For this reason, it is crucial to understand both the mechanisms used by the infectious agents (virus and/or bacteria) to destroy the ependymal lining, as well as the inflammatory process that follows.

A single intracerebroventricular injection of *Clostridium perfringens* neuraminidase (NA) within the lateral ventricle of rats induces the massive detachment and death of the ependymal cells, followed by acute inflammation in ventricles and meninges and obstructive hydrocephalus due to Sylvius aqueduct stenosis [21]. The ependymal cell death occurs immediately after the injection and prior to inflammation, indicating that it is caused by the presence of NA in the CSF and not by inflammatory cells. The NA catalyzed sialic acid removal from the ependymal cell glycocalyx brings about ependymal death by an unknown mechanism [21].

NA is an exo-glucosidase that removes terminal sialic acid from glucidic chains, preferably those joined by α2–3 (α2–3 > α2–8 = α2–6) linkages. In most glucidic chains attached to proteins, galactose is the sub-terminal sugar residue [22], which becomes exposed after NA action. Thus, when NA acts on the ependymal lining, the sialic acid cover is replaced by a galactose cover.

We hypothesize that NA directly provokes ependymal cell death because (1) it removes the sialic acid protection from the cell surface and (2) it activates the complement system in the CSF. Both events lead to the deposition of complement activation products, including the membrane attack complex (MAC), onto the cellular surface resulting in cell damage or death.

The complement system is a humoral innate defensive mechanism formed of about 40 plasma- and membrane-bound proteins that can be activated by three different pathways: the classical pathway, which requires the participation of immunoglobulins; the alternative pathway, activated by “foreign” surfaces; and the lectin pathway, initiated by certain sugars. All of them converge at a common step, the cleavage of the C5 component into C5a and C5b. This event triggers the assembly of factors C6, C7, C8, and C9 that form the MAC on the target cells [23].

The above-mentioned hypothesis is based upon the following facts:

1. All the components of the classical and alternative complement pathways are present in the CSF to provide protection to the CNS against infectious agents, starting an inflammatory reaction and aiding the removal of cellular debris [24–26].
2. Ependymal cells bear at their surface proteins that regulate the complement system, such as CD55 and CD59, which are upregulated upon infections like meningitis [27]. This fact supports the capacity of ependymal cells to modulate complement-mediated injuries triggered by cerebral insults.
3. Sialic acid is abundant on the surface of ependymal cells [21]; it protects ependymal cells by (i) increasing factor H affinity, which inhibits the activation of complement by the alternative pathway and (ii) preventing the deposition of MAC [28, 29].
4. Sialic acid removal from human tumor cells and red blood cells renders them vulnerable to alternative pathway activation and the MAC [30, 31]. Similarly, sialic acid in the bacteria lipopolysaccharides inhibits complement attack and formation of MAC on bacterial cells, thus contributing to bacterial virulence [32].
5. NA can directly activate the alternative complement pathway, both in the fluid phase and on the cell surface [33].

The aim of this work was to analyze to what extent the complement system is responsible for the death of ependymal cells induced by NA. Therefore, we studied in an in vivo model the formation of MAC deposits onto the cells damaged by NA and the activation of the different complement pathways in the NA-injected ventricle. We also confirmed in vitro the ability of NA to directly activate complement. Finally, we tested the effects of absence or inhibition of MAC formation in vivo on the death of ependymal cells upon treatment with NA.

## Methods

### Animals

Adult male Wistar rats (Charles River Laboratories, Barcelona, Spain) (body weight 250–300 g) were used in this study. In addition, a strain of rats deficient in the C6 component of complement that is unable to generate membrane attack complex [34, 35] was used. All animals were housed under a 12-h light/dark cycle with food and water available ad libitum. Animal procedures were performed according to the European Union (86/609/EEC) and Spanish (RD 1201/2005) legislations. Animal care and experimental procedures were approved by the Animal Experimentation Ethics Committee of the University of Málaga (ref. number 2012-0013).

### Intracerebroventricular injection

The animals were anesthetized with 2,2,2-tribromoethanol (0.2 g/kg bw, Fluka Chemika) and positioned in a stereotaxic frame. NA from *C. perfringens* (Roche Diagnostics, 11 585 886 001) dissolved in 0.9 % sterile saline (25 mU/μl) was administrated by stereotaxic surgery into the right lateral cerebral ventricle (intracerebroventricular (icv)). The coordinates from Bregma used were antero-posterior −0.5 mm, medio-lateral −1.4 mm, and dorso-ventral −3.5 mm. A single dose of 500 mU (20 μl) of NA was injected with the aid of a pump at a rate of 2 μl/min during 10 min. Control rats were injected with saline or with heat-inactivated NA [36]. Some animals were sacrificed immediately after NA injection (time 0 h), and the rest were recovered from anesthesia and sacrificed at 2 h, 4 h, 12 h, 24 h, 2 days, 4 days, 7 days, 15 days, 30 day, 3 months, and 6 months after the administration of NA.

### Inhibition of the complement component C5

In order to obtain both systemic and ventricular inhibition of complement component C5, a strategy consisting of the injection of a blocking monoclonal anti-rat C5 monoclonal antibody (C5-inh; clone 18A10, Alexion Pharmaceuticals Inc. [37, 38]) was employed. The treatment followed published protocols [39]; an initial intravenous injection of the antibody (20 mg/kg bw) was followed 6 h later by an intraperitoneal injection of the same antibody (10 mg/kg bw). According to the published data, this protocol achieved a 50–60 % inhibition of C5 activity for up to 12 h after the first injection. The highest inhibition occurs approximately 1 h after the second injection. At this time of maximal systemic inhibition, the antibody was also injected icv to obtain a ventricular inhibition of C5. The inhibitor was icv injected along with NA (20 μl of a mixture of NA 25 mU/μl and C5-inh 0.38 μg/μl) following the same parameters outlined above.

### Obtaining and culture of ventricular wall explants

The brains of the euthanized rats were removed, and ventricular wall explants were obtained following the protocol described elsewhere [40]. The explants were washed in DMEM-F12 medium (Gibco) and incubated for 2 h (37 °C, 5 % CO_2_) in the same medium supplemented or not with neuraminidase (5 mU/μl) or rat serum (50 % *v*/*v*) as a source of complement factors. Five replicates of each experimental situation were performed. The explants were finally fixed by immersion in Bouin’s fixative for 24 h and processed for histological sectioning.

### Obtaining and culture of isolated ependymal cells

Ventricular wall explants were used in order to obtain pure isolated ependymal cells [40]. Briefly, the explants were sequentially incubated in (1) HBSS without calcium and magnesium (Gibco); (2) TrypLE Express ™ solution (Gibco); (3) αMEM (Gibco) supplemented with 0.2 % Pluronic F-127 (Sigma), 0.3 % D-glucose (Sigma), and 0.01 M HEPES (Sigma; for details of incubation times and temperatures, see [40]). During the last incubation, the cells detach from the explant and are released to the medium. They were then harvested by centrifugation (300×*g*, 10 min) and resuspended in supplemented αMEM.

Isolated ependymal cells were placed in individual wells in a final volume of 1.6 ml and at a density of 10^4^ cells/ml in αMEM with D-glucose and HEPES. According to each experimental situation, the medium was supplemented with NA (5 mU/μl), serum (50 % v/v) as a source of complement factors, and/or C5-inh (0.4 μg/μl; Alexion Pharmaceuticals Inc.).

Cell viability was then assessed by trypan blue staining. Cell samples (200 μl, about 2000 cells) from each well were taken at different times: 0 min (before adding the treatments), 5, 10, 20, 30, 40, 50, and 60 min. Each 200-μl cell sample was mixed with 200 μl of a 0.4 % trypan blue solution (Sigma), incubated (5 min), and centrifuged (500×*g*, 5 min). The cell pellet obtained was resuspended in 100 μl 4 % of paraformaldehyde fixative and placed on a slide for counting under an optical microscope. The experiment was performed in triplicate.

### Histological procedures

The animals were anesthetized with 2,2,2-tribromoethanol and transcardially perfused with saline followed by Bouin’s fixative. Brains were removed and immersed in the same fixative for 24 h at room temperature (RT) and later embedded in paraffin wax. Cultured explants were fixed by immersion in Bouin’s fixative for 24 h and later embedded in paraffin as well. Seven-micrometer sections were obtained from each brain/explant and mounted on poly-l-lysine-treated slides. Series of sections along the brain region of interest (from bregma −0.3 to −1.2 mm approximately) were obtained. Some brains were harvested unfixed to obtain frozen sections. Fresh brains from the euthanized rats were removed, washed in 0.1 M phosphate-buffered saline (PBS), embedded in Tissue-Tek OCT™ Compound (Sakura), and frozen on a metal plate cooled with liquid nitrogen. Ten-micrometer sections were obtained with a cryostat and immediately fixed in cold acetone for 10 min, as described [41] for complement factor preservation. Hematoxylin-eosin staining was applied to counterstain the sections.

### Lectin binding

The peanut (*Arachis hypogaea*) agglutinin (PNA, Vector) was chosen for its exclusive affinity for terminal residues of galactose, which are the sub-terminal sugar residues in complex-type glycoproteins. After the removal of terminal sialic acid, galactose is exposed as a terminal residue, and the PNA binding sites arise. To check for the removal of sialic acid by the action of NA, the sections from the explants were incubated in 3 μg/ml peroxidase-labeled PNA in 0.1 M PBS for 1 h at RT. The peroxidase activity was detected with 0.1 M PBS containing 0.05 % diaminobenzidine (DAB, Sigma) and 0.03 % hydrogen peroxide (Merck).

### Immunohistochemistry

Immunohistochemistry was carried out on deparaffinized tissue sections using either immunoperoxidase or immunofluorescence techniques. The primary antibodies used were rabbit anti-rat C5b-9 (1:200, Abcam, ab55811); rabbit anti-rat C9 (1:100, provided by Prof. B. P. Morgan, Cardiff University); and mouse anti-rat vimentin (1:1000, Sigma, V6630). The secondary antibodies used were biotinylated goat anti-rabbit IgG (H+L) (1:1000, Pierce, 31820); Alexa fluor® 488 goat anti-rabbit IgG (H+L) (1:1000, Molecular Probes, A11070); Alexa fluor® 594 donkey anti-mouse IgG (H+L) (1:1000, Molecular Probes, A21203). All antibodies were diluted with PBT buffer (0.3 % bovine serum albumin, 0.3 % Triton X-100 in PBS pH 7.3). The primary antibodies were incubated overnight at RT. The secondary antibodies were incubated for 60 min at RT. Negative controls for the immunostaining consisted in equivalent sections are subjected to the same protocol but omitting the primary antibody.

Prior to the overnight incubation with the primary antibody, the sections stained with the immunoperoxidase procedure were incubated in 3 % hydrogen peroxide and 10 % methanol in phosphate buffer (PB) 0.1 M to quench endogenous peroxidase activity. In such sections, biotinylated secondary antibody was used, along with the avidin-biotin-complex (ABC) amplification method. The ABC reagent was prepared according to the manufacturer’s instructions (Thermo Fisher Scientific) and incubated for 30 min. Peroxidase activity was revealed with 0.05 % DAB and 0.03 % hydrogen peroxide. All the incubations were performed in a moist chamber at RT.

For immunofluorescence, Alexa fluor® 488- or Alexa fluor® 594-conjugated secondary antibodies were used, and the DNA binding dye 4,6-diamidine-2-phenylindole dihydrochloride (DAPI) (0.1 μg/ml) was added to the secondary antibody solution. Coverslides were mounted with buffered glycerol containing 10 % of the anti-fading agent Mowiol 4-88 (Calbiochem/EMD Chemicals). The sections were then observed under a confocal microscope (Leica, SP5 II).

### Obtaining and activation of serum and CSF

Rat blood was drawn by puncture of the tail vein, under sterile conditions and kept cold to preserve the activity of the complement system. Once coagulated, serum was prepared by centrifugation (1500×*g*, 10 min, 4 °C).

To obtain CSF, anesthetized rats were placed in a stereotaxic apparatus with the head tilted at an angle of 75° to the dorsal spine. A needle connected to a cannula was introduced through the cranium-atlas joint into the *cisterna magna*, at a depth of about 7 mm. CSF was removed with a pump (KD Scientific, model KDS-210-CE) connected to the syringe at a rate of 2 μl/min in 20 μl fractions, with pauses of 5 min between fractions, until a total volume of 80–100 μl was obtained. The samples were immediately placed in ice to preserve complement activity. We checked that the CSF was clear and not contaminated with blood. Extracted CSF was collected in a sterile tube and stored at −80 °C until use.

### Obtaining of ependymal epithelium extracts

The extracts were obtained from the explants of the ependymal wall in order to analyze the presence of certain proteins by Western blot. The explants obtained were weighed, homogenized in ice, and 30 μl of protein extraction buffer (Complete Mini (Roche) in 20 mM Tris-HCl, 100 mM NaCl, 0.2 % Triton X-100) were added per milligram of tissue. Sonication was applied to further homogenize. The homogenate was then centrifuged (5600×*g*, 5 min, 4 °C) and the supernatant recovered. Finally the total protein concentration was determined by the bicinchoninic acid method (BCA Protein Assay Kit, Pierce), prior to analysis in Western blot.

### Immunoprecipitation

In order to enrich the samples for subsequent detection by Western blot of the proteins C1q and mannose-binding lectin (MBL), immunoprecipitation was applied to the serum and ependymal epithelium extracts. The procedure was based on that suggested by the manufacturer of the Mouse TrueBlot® Set IP-Beads kit (Rockland). Briefly, after pre-washing of the beads and the pre-clearing of the samples, to each aliquot (500 μl of serum or extract diluted 1 mg/ml in PBS) was added 3 μg of primary antibody (mouse anti-C1q, Abcam 71940 or mouse anti-MBL, Abcam 23461) and 50 μl of pre-washed beads. The samples were incubated 2 h at 4 °C on rotation, and then centrifuged (2500×*g*, 3 min, 4 °C). The supernatant was removed and the beads were washed three times with 500 μl of RIPA buffer (50 mM Tris-HCl, 150 mM NaCl, 1 mM EDTA, 1 % NP-40, and 0.25 % sodium deoxycholate). After the last wash, the beads were resuspended in 50 μl of gel loading buffer, heated at 95 °C for 10 min, and centrifuged (10,000×*g*, 5 min), and the supernatant obtained was subjected to polyacrylamide gel electrophoresis (PAGE).

### Western blot

Western blot was used to identify the deposition of C1q and MBL on the ependyma of NA-injected rats. For this purpose, the serum and ependyma samples were subjected to immunoprecipitation as described above.

On the other hand, the activation of complement in serum and CSF samples was analyzed by monitoring the fragmentation of C3 by Western blot. In this case, serum and CSF 50 μl samples were incubated with 5 μl of zymosan 0.1 g/ml (positive control of complement activation), 5 μl of NA (50 mU/μl), or 5 μl of 0.9 % saline (negative control of complement activation) for 1 h at 37 °C. Afterwards, they were centrifuged (9000×*g*, 10 min), and the supernatant was recovered for PAGE.

The protein samples mentioned above were resolved in 7.5 % SDS-PAGE (for the detection of C3) or 15 % SDS-PAGE (for the detection of C1q and MBL) in the presence of 2-β-mercaptoethanol (reducing conditions). After electrophoresis, the proteins were transferred to a PVDF membrane. The membranes were blocked with 5 % skimmed milk and 1 % Tween-20 in PBS and incubated overnight at RT with the primary antibodies mouse anti-rat C3 (1:140, C3.30, Prof. B. P. Morgan, Cardiff University); rabbit anti-C1q (1:100, Hycult, HP8021); and mouse anti-MBL (1:500, Abcam, 23461). As a negative control of the primary antibody binding, a membrane corresponding to a serum sample was treated alike but excluding the primary antibody. The corresponding horseradish peroxidase-conjugated secondary antibody (anti-mouse TrueBlot®, 1:1500, Rockland, 18-8817-31; anti-rabbit IgG, 1:1000, Sigma, A8275) was probed for 1 h at RT, and detection of the antibody binding was determined by chemiluminescence (Clarity™ Western ECL, Bio-Rad). The chemiluminescence was recorded using a camera Molecular Imager (Bio-Rad, ChemiDoc™ XRS+) and Image Lab 4.0 (Bio-Rad) software.

### Quantification of ependymal disruption

In order to quantify the severity and the extension of the ependymal alterations, the percentage of intact, damaged, and disrupted ependyma was measured on histological sections immunostained with anti-vimentin. Four different experimental situations were analyzed: (i) sham-operated wild-type rats, (ii) NA-injected wild-type rats, (iii) NA-injected C5-inhibitor-treated wild-type rats, and (iv) NA-injected C6-deficient rats. All animals were sacrificed 2 h post-injection of NA. Three rats from each experimental situation were used. The percentage of intact, damaged, and disrupted ependyma was measured on three histological sections from each animal. The image analysis software ImageJ (NIH) was used.

### Statistical analysis

Differences between the percentage of living cells in the different treatments were evaluated by ANOVA (significance level of 0.05), followed by a Tukey’s post hoc test with a level of significance of 0.05. The values measured at time 0 were considered 100 %. The software SPSS® Statistics 20 (IBM®) was used for this analysis.

## Results

### Membrane attack complex deposition onto ependymal cells of NA-treated rats

The initial approach to investigate the potential role of the complement system in ependymal cell death caused by icv injected NA was to localize the MAC on the apical surface of the ependymal cells. This complex is composed of the proteins C5b, C6, C7, C8, and several units of C9 [42]. The immunocytolocalization of MAC was performed by using two different antibodies which recognized, respectively: (i) an epitope of C5b-9 not present in non-polymerized fragments and (ii) an epitope of C9 located in the polymer forming the transmembrane pore [43] (see “Methods”).

**Fig. 1:**
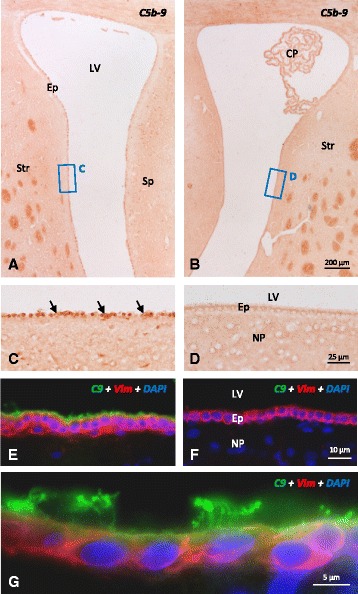
Detection of C5b-9 complex and C9 on the ependymal epithelium of rats injected with NA. **a**–**d** Immunohistochemistry using anti-C5b-9 showing the injected (**a**, **c**) and the contralateral (**b**, **d**) ventricles. Two hours after NA injection, a positive label could be observed on the ependymal surface of the injected ventricle (**c**), which was absent in the contralateral one (**d**). **e**–**g** Cryostat 10-μm sections double labeled with anti-C9 (*green*), anti-vimentin (*red*), and the nuclear stain DAPI (*blue*). The ependymal cells of the injected ventricle showed C9 deposits on the apical surface and cilia (**e** and **g**) that were not present in the contralateral ventricle (**f**). *LV* lateral ventricle, *DAPI*, 4,6-diamidine-2-phenylindole dihydrochloride, *Ep* ependyma, *NP* nervous parenchyma, *CP* choroid plexus, *Str* striatum, *Sp* septum. (*Arrows*) Damaged cells

Anti-C5b-9 labeled the apical membrane and cytoplasm of most ependymal cells in the NA-injected ventricle (2 h after injection) (Fig. 1a, c), a staining that was absent in the contralateral non-injected ventricle (Fig. 1b, d). C5b-9-positive ependymal cells presented an irregular shape and seemed to be detached from the ventricular wall (Fig. 1c), compared to the ependymal cells in the contralateral ventricle which kept a normal epithelial morphology (Fig. 1b, d). A similar result was obtained with anti-C9 antibody. Double immunofluorescence showed a positive immunostaining at the apical surface and cilia of ependymal cells only in the injected ventricle (Fig. 1e, g), but not in the contralateral one or in non-injected rats (Fig. 1f).

### NA activates complement component C3 in vitro

**Fig. 2:**
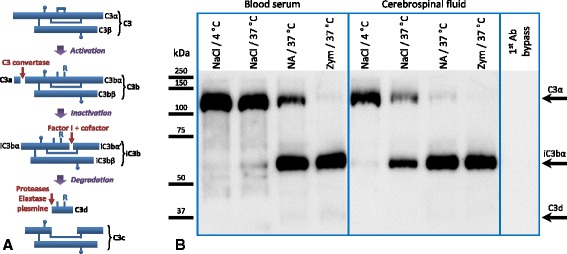
Neuraminidase induces C3 activation in vitro. **a** Scheme showing the activation, deactivation, and degradation of the complement factor C3, based on [44, 45]. The anti-C3 antibody used for the immunoblot detects C3 α chain (113 kDa) before activation, and the iC3b α’ chain fragment (62.5 kDa) generated after activation [46]. **b** Blood serum andcerebrospinal fluid (CSF) samples were used as the source of factor C3. They were incubated with NA at 37 °C for 1 h (NA/37 °C), and then analyzed by immunoblot with anti-C3. Controls included: incubation in saline (NaCl) without NA, both at 37 °C (NaCl/37 °C) and at 4 °C (NaCl/4 °C); a positive control of complement activation with zymosan (Zym/37 °C). Complement activation correlates with fading of the C3 α band and concurrent appearance of iC3b α’ band. As expected, activation occurred in the positive controls (Zym/37 °C) but not in the negative controls (NA/4 °C), both in serum and CSF samples. NA was also able to activate complement (NA/37 °C), although not as much as zymosan, as the appearance of the iC3b α’ band is delayed. A partial activation was detected in the negative control NaCl/37 °C, which can be ascribed to auto-activation of complement when it is maintained for 1 h at 37 °C. The specificity of the anti-C3 antibody was proved by omitting the primary antibody during the immunoblot procedure (1st Ab bypass)

Western blot is a sensitive technique that test the activation of the complement system in vitro. C3 gives rise to several fragments during its activation, deactivation, and degradation process [44, 45] (Fig. 2a). An anti-C3 monoclonal antibody recognizing C3 α chain (113 kDa) before activation, and the iC3b α’ chain fragment (62.5 kDa) generated after activation [46], was used in immunoblot to investigate whether NA was able to induce the activation of complement in serum and CSF samples. When serum or CSF samples were incubated with NA, the iC3b α’ fragment was formed (Fig. 2b, lanes “NA/37 °C”), also seen when the samples (blood serum or CSF) were incubated with zymosan, a complement activator used as a positive control in this experiment (Fig. 2b, lanes “Zym/37 °C”). In conditions where complement was not activated, the iC3b α’ fragment was not observed (Fig. 2b, lanes “NaCl/4 °C”). We did observe some activation (presence of iC3b α’ fragment) in samples without NA but incubated at 37 °C (Fig. 2b, lanes “NaCl/37 °C”), compared to the same samples maintained at 4 °C. We infer that this is due to auto-activation of complement and was always less than the activation triggered by NA (Fig. 2b, lanes “NA/37 °C”).

### Identification of the complement pathways activated by NA

The initiation of the classical pathway of complement is mediated by the binding of C1 to IgM or IgG molecules previously bound to their antigens. The desialylation of the ependymal epithelium by NA might expose epitopes recognized by immunoglobulins, therefore allowing the assembly of C1 and triggering the classical pathway activation. We first tried to detect immunoglobulin deposits on the ependymal cells of the rats injected with NA by immunohistochemistry using anti-IgG antibodies. However, no IgG deposits were detected (not shown).

**Fig. 3:**
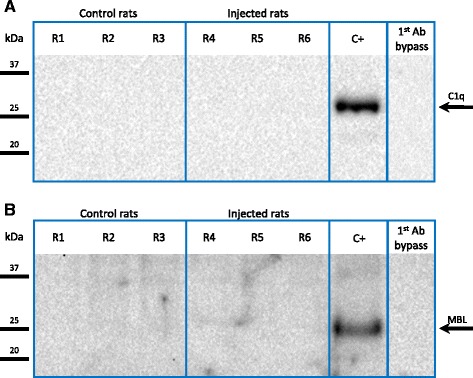
Western blot detection of C1q and MBL in ependymal wall extracts. Protein extracts were prepared from the lateral wall of rats injected with NA (*R4*–*R6*) and non-injected control rats (*R1-R3*). In order to increase the amount of target proteins (*C1q* and *MBL*) loaded in the gel, they were immunoprecipitated from the extracts prior to the Western blot. Neither C1q (**a**) nor MBL (**b**) were detected in ependymal wall extracts, regardless of the treatment with NA. Positive controls (*C+*) consisted in rat serum samples are subjected to the same procedure; the specificity of the antibodies was checked by omitting the primary antibodies (1st Ab bypass)

Next, the presence of C1q (a C1 complex component) on the ependyma of the NA-injected rats was searched by immunohistochemistry (not shown) and by Western blot (Fig. 3a). C1q was not detected in any case. A 26-kDa band corresponding to the expected molecular weight of C1q A chain was detected in the normal rat serum samples used as the positive control (Fig. 3a, lane “C+”).

The sialidase activity of NA on ependymal cells results in the exposure of galactose residues in N-linked glycoproteins [47]. These residues pose a potential binding site for MBL, which activates the complement lectin pathway. The presence of MBL on the ependymal surface of the NA-injected rats compared to non-injected control rats was analyzed by immunohistochemistry and immunoblot. The anti-MBL antibody did not show the presence of MBL on the ependymal surface by immunohistochemistry (not shown) nor in the ependyma extracts by Western blot (Fig. 3b). However, a band of 25 kDa corresponding to the MBL monomer was observed in the rat serum samples used as the positive control (Fig. 3, “C+” lane).

### The complement system enhances NA-induced ependymal cell damage in vitro

Ventricular wall explants were used to determine the effect of NA on ependymal cells in the presence or absence of complement. Cell viability was evaluated by monitoring the ciliary movement at different times during a 2-h period. Serum was used as a source of complement. As shown in Table 1, ciliary movement was unaltered in control explants incubated in culture medium (CM) as well as in explants incubated in the presence of complement (CM + serum). However, cilia beating decreased after 1 h incubation with NA, either in the presence (CM + NA + serum) or absence (CM + NA) of complement.

**Table 1:** Number of explants showing cilia movement, out of five explants per condition

Culture media	Pre	0 h	1 h	2 h
CM	5	5	5	5
CM + NA	5	5	2	0
CM + serum	5	5	5	5
CM + NA + serum	5	5	3	1

**Fig. 4:**
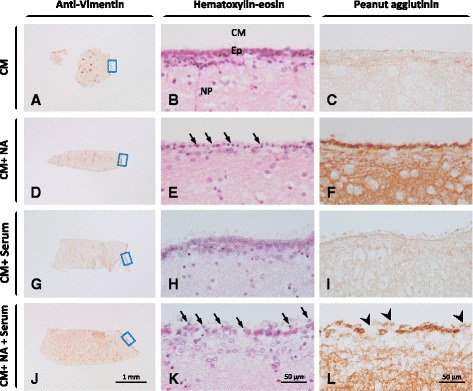
In vitro effects of NA and complement on the ependymal epithelium. Explants from the septal side of the lateral ventricle wall were placed in culture and treated with NA and/or complement for 2 h. Rat serum was used as a source of complement. The ependyma in the explants was then evaluated by histology. Anti-vimentin allowed the location of the ependyma in the explants (**a**, **d**, **g**, **j**). The *squared* regions are those magnified in the hematoxylin-eosin staining (**b**, **e**, **h**, **k**). The sialic acid removal by NA was confirmed by binding of the lectin PNA (peanut agglutinin; **c**, **f**, **i**, **l**). Ependymal damage occurred in explants treated with NA (**e**, **k**) and was worsened by the presence of serum (**k**). The concurrence of NA and serum resulted in the loss of epithelium integrity, where gaps could be seen (*arrowheads* in **l**). Serum alone did not produce such damage (**e**). *CM* culture medium, *NA* neuraminidase, *Ep* ependyma, *NP* nervous parenchyma. (*Arrows*) Damaged ependymal cells

To further analyze the results of the previous experiment, the explants were fixed for histological evaluation. The PNA lectin was used to confirm the sialidase action of NA, and anti-vimentin immunohistochemistry to confirm the presence of ependymal cells in the explants. All NA-treated explants were PNA-positive (Fig. 4f, l), confirming sialic acid removal. The hematoxylin-eosin staining revealed that explants incubated with culture medium alone (CM, Fig. 4a–c) displayed an unaltered ependymal cell layer with cells that were slightly eosinophilic. Similar results were observed in the explants treated with culture medium and complement (CM + serum, Fig. 4g–i). However, the ependymal cells in the explants incubated with NA (CM + NA, Fig. 4d–f) showed signs of cell damage, such as a smaller cytoplasm and pycnotic nuclei. When complement was added in addition to NA (CM + NA + serum, Fig. 4j–l), the ependymal cells lost lateral cell-cell contacts and, therefore, the ependymal epithelium appeared disorganized and displayed occasional discontinuities. Hence, the experimental condition CM + NA + serum was the one causing most damage, which suggests that serum and NA acted synergistically to harm ependymal cells.

**Fig. 5:**
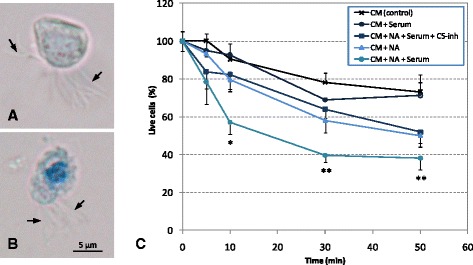
In vitro effects of NA and complement on ependymal cell viability. Pure ependymal cells were isolated from adult rats and placed in culture in the presence of NA and/or complement. Rat serum was used as a source of complement. **a**, **b** The viability of cells was monitored by the exclusion of trypan blue stain; the living ependymal cells excluded the dye (**a**) while the dead cells stained blue (**b**). *Arrows* point to the cilia. **c** The graph shows the percentage of live cells at different time points relative to living cells at time zero. The data are mean ± SEM of three independent experiments. In the presence of NA and serum, there was a steep decline of the viability as soon as 10 min after the start of the exposure, which continued dropping up to 40 % survival after 30 min. A milder decline in viability was also observed in cells treated with NA alone with no serum. The involvement of the complement in facilitating NA-induced cell death was tested by using a complement blocking antibody (C5-inh), which completely reversed the survival rate to the values obtained with NA alone. *CM* culture medium, *NA* neuraminidase, *C5-inh* C5 inhibitor; *P < 0.05; **P < 0.01 compared to the control CM

A similar experimental design was performed but in this case using a primary culture of pure ependymal cells obtained from adult rats [40]. Viability of ependymal cells was assessed with the vital stain trypan blue. The number of viable ependymal cells at the beginning of the experiment was considered as 100 %. Viability was measured at different time points after the addition of NA and/or serum (Fig. 5). Viability decreased with time in all culture conditions, but the addition of NA further reduced it, and this loss of viability was further accelerated when serum was also present. In this condition (NA + serum) viability dropped to 39.6 ± 3.5 % in just 30 min. The addition of a complement inhibitor (C5-inh) partially prevented this decrease in viability (64.0 ± 4.9 %), demonstrating that complement present in serum is responsible for the drop in viability.

### C5 inhibition in NA-treated rats does not prevent ependymal damage but decreases ependymal disruption

The proteolysis of the complement factor C5 yields the fragment C5b, which is the first component responsible for the initiation of the formation of MAC. Therefore, C5 proteolysis is an essential step for MAC assembly [23]. It has been demonstrated that the systemic inhibition of C5 proteolysis by treatment with anti-C5 antibodies impedes the formation of MAC [37, 38].

**Fig. 6:**
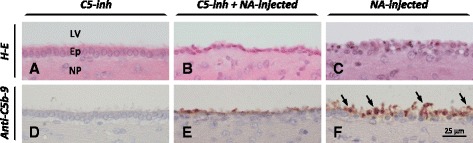
Effect of NA on the ependyma of rats under systemic inhibition of C5. A group of rats were treated with a monoclonal antibody for the systemic inhibition of C5. Some of them were icv injected with NA (C5-inh + NA; **b**, **e**) while others were not injected to serve as controls (C5-inh; **a**, **d**). The control group for NA-dependent ependymal damage was injected with NA but not systemically inhibited (NA; **c**, **f**). The ependyma of the injected ventricle was histologically evaluated, and the formation of the complement complex C5b-9 (MAC) studied by immunohistochemistry. In the rats under systemic inhibition of C5, NA produced some damage to the ependymal epithelium; the cells appeared flattened and presented a condensed pycnotic nucleus (**b**). However, the deposits of MAC were modest (**e**). These changes were mild compared to those provoked by NA in non-inhibited rats (**c**), where the ependymal epithelium was disrupted and MAC deposits were conspicuous (**f**). Rats C5 inhibited but not injected with NA showed an unaltered ependyma (**a**), and no MAC deposits at all (**b**). *LV* lateral ventricle, *Ep* ependyma, *NP* nervous parenchyma

To test the participation of MAC in the ependymal cell death due to NA, the rats were systemically and ventricularly inhibited with anti-C5 and then treated with an icv injection of NA. Histological study of the ependymal epithelium 2 h after the injection of NA revealed alterations of ependymal cells, which acquired a flattened shape and pycnotic nucleus. However, the ependymal epithelium was not seriously disrupted (Fig. 6b; Fig. 8a; 11.3 ± 2.4 % disrupted ependyma). In contrast, epithelial integrity was lost in rats treated with NA without C5 inhibition (Fig. 6c; Fig. 8a; 53.5 ± 8.5 disrupted ependyma). The ependymal cells in the contralateral ventricle of NA-treated rats appeared unaffected, as did those in rats treated with anti-C5 but not injected with NA (Fig. 6a). The immunohistochemical analysis with anti-C5b-9 showed the presence of MAC deposits on the surface of the damaged ependyma; MAC deposits were less prominent in the C5-inhibited rats (Fig. 6e–f).

### NA-induced ependymal cell disruption is reduced in C6^−/−^ rats

**Fig. 7:**
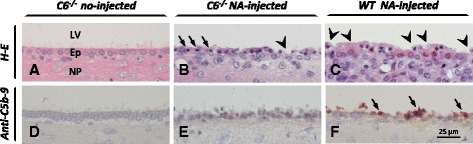
Effect of NA on the ependyma of C6-deficient rats. The damage caused by icv-injected NA on the ependyma was studied in a strain of rats deficient for the complement factor C6 (**b**, **e**) 2 h after the injection of NA. Normal wild-type (WT) rats served as control for the NA-induced ependymal damage (**c**, **f**). The formation of MAC deposits was evaluated by immunohistochemistry (**d**–**f**). The injection of NA did not induce MAC assembly onto the ependyma of C6^−/−^ rats (**e**). However, some ependymal cells presented a slightly condensed nucleus (*arrows* in **b**), while the epithelium appeared with some minor disruptions (*arrowhead* in **b**) compared to that of control wild-type rats. These changes were minor compared to those observed in wild-type rats treated with NA (**c**), where the epithelium appeared disorganized (*arrowheads* in **c**), and MAC deposits were abundant (*arrows* in **e**). Ventricular walls from non-injected C6-/- rats (**a** and **d**) did not display any anomaly and were comparable to non-injected WT rats (data not shown). *LV* lateral ventricle, *Ep* ependyma, *NP* nervous parenchyma, *H-E* hematoxylin and eosin staining

**Fig. 8:**
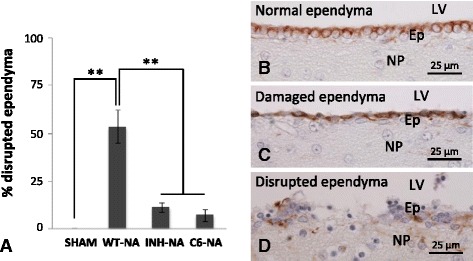
Quantification of ependymal epithelium disruption in different experimental conditions. **a** The graph shows the proportion of disrupted ependyma relative to the total ependymal perimeter measured in histological sections. Four experimental situations were analyzed: sham-operated rats (*SHAM*), NA-injected wild-type rats (*WT-NA*), NA-injected C5-inhibitor-treated rats (*INH-NA*), and NA-injected C6-deficient rats (*C6-NA*). Photographs show the appearance of intact (**b**), damaged (**c**), and disrupted (**d**) ependymal epithelium immunostained with anti-vimentin. NA-injected WT rats display 53.5 ± 8.5 % of ependymal epithelium disruption. However, in rats with the complement system inhibited (IHN-NA and C6-NA), this proportion decreases significantly (11.3 ± 2.5 and 7.1 ± 3.3, respectively). *Bars* represent the mean ± SEM. Different treatments were compared by one-way ANOVA (*F*_(3,11)_ = 25,872, *P* < 0.05) and Tukey’s range test. **P < 0.01. *LV* lateral ventricle, *Ep* ependyma, *NP* nervous parenchyma

In C6^−/−^ rats the complement factor C6 is absent; hence, MAC assembly is not possible. The ependymal cells of the C6-deficient rats injected with NA displayed some morphological alterations (arrows in Fig. 7b) but only a minor disorganization of the epithelium (arrowhead in Fig. 7b; Fig. 8a, 7.1 ± 3.2 % disrupted ependyma), compared to NA-injected wild-type rats, where the epithelium was markedly disrupted (arrowheads in Fig. 7c; Fig. 8a; 53.5 ± 8.5 % disrupted ependyma). C5b-9 deposits were present on the ependyma of wild-type NA-injected rats but, as expected, they were not detected on the damaged ependyma of C6^−/−^ mutant rats (Fig. 7e–f), suggesting that MAC assembly is a major contributor to but is not the sole cause of ependymal injury triggered by NA.

## Discussion

A single icv injection of NA in the lateral ventricle of rats results in the death of ependymal cells within 1 h after the injection, followed by long-term hydrocephalus [21]. This method of causing a lesion to the ependyma has been used here and by other authors [47–50], rendering similar outcomes with respect to ependymal destruction and the subsequent inflammatory reaction [9]. Despite the long history of the use of this model, the precise mechanism of ependymal cell death after the removal of sialic acid by NA is unknown. Ependymal death occurs immediately after injection of NA and prior to inflammatory cell infiltration. Therefore, inflammation does not seem to be responsible for the observed cell death, and a more direct action of NA should be considered. Several lines of evidence support a role for the complement system: (i) the components of the complement system are present in the CSF [24–26] (ii) cells deprived of sialic acid at their surface are more vulnerable to complement attack [29, 31, 51]; and (iii) the complement system can be activated by NA [33]. Hence, this study was designed to test the involvement of the complement system on the death of ependymal cells induced by NA.

### Neuraminidase activates the alternative pathway of complement and induces MAC deposition onto ependymal epithelium

Immunohistochemistry demonstrated the presence of MAC on the surface of the ependyma 2 h after the injection of NA. It is known that several cell types in the nervous system can produce proteins of the complement system [25, 52–54]. However, we can rule out that the observed staining of ependymal cells is a result of synthesis of C9 because (i) the antibodies used are specific for the activated/polymerized forms of the proteins and (ii) the label was more intense in those cells that appeared more damaged. The ependymal identity of the labeled cells could be established by their location, morphology, and the presence of a tuft of cilia. Also, C9 staining colocalized with vimentin, an intermediate filament expressed by ependymal cells.

The activation of complement that promotes MAC assembly onto the ependyma could be ascribed to (1) the direct action of NA on complement factors present in the CSF and/or (2) the suppression of the regulatory factors that restrict complement activation, such as surface sialic acid [31], factor H [29], or CD59 [55, 56]. Sialic acid residues present in the bacterial capsule polysaccharides represent a virulence factor, as they help to evade complement activation and formation of MAC [32]. Also, it has been reported that sialic acid removal from the surface of human cancer cells, red blood cells, and other cell types makes them more prone to MAC deposition [29, 31, 51]. Factor H is a soluble factor that binds to sialic acid in cell membranes [29]. It inhibits the formation of C3/C5 convertase complexes and arrests the amplification of the activation signal. Factor H effectiveness is considerably decreased when the target cell surface is devoid of sialic acid [55, 57, 58]. CD59 is a complement activation inhibitor which prevents the formation of MAC by interacting with C5b 8 and blocks C9 polymerization [55, 56]. CD59 itself bears sialic acid, so NA could jeopardize CD59 inhibitory action. Although it has been shown that sialic acid removal by NA does not affect CD59 function in erythrocyte membranes [59], this fact has not been tested in CNS cells. In the present report, neither the factor H nor CD59 was analyzed in ependymal cells of NA-injected rats, which would be of interest in future works.

Western blot analysis revealed the presence of fragments resulting from the activation of C3 in CSF and serum samples exposed to NA. This could be the result of the action of NA on C3 directly or on other factors involved in the formation of soluble C3 convertase complexes. Although the incubation of serum and CSF samples was carried out in the absence of cell membranes, it has been shown that the assembly of C3 convertase complexes is feasible even if no membrane attachment occurs [60, 61].

On the other hand, no deposits of C1q or MBL could be detected on the ependymal surface of the NA-injected ventricle, neither by immunohistochemistry (not shown) nor by Western blot. All the above evidences suggest that, as Fujita [33] previously proposed for human complement, NA accelerates the activation of rat complement through the alternative pathway, with no participation of C1 from the classical pathway or MBL from the lectin pathway.

### Inhibition or blockade of MAC formation reduces NA-induced ependymal damage

To assess the role of the complement system, and specifically the MAC, in ependymal destruction caused by NA, the action of this enzyme was evaluated under a situation of partial inhibition or complete blockade of MAC formation. For this purpose, two different in vitro assays were developed, one using pure ependymal cell primary cultures and the other explants from the wall of the lateral ventricle. In both experiments, NA alone resulted in damage of the ependymal cells, although damage was greater when the complement system was also present. Therefore, NA by itself is able to impair the ependymal cells inducing morphological alterations, nuclear pycnosis and loss of cilia motility. Cilia retraction in ependymal cells after exposure to NA has been previously described [49]. However, complement further contributes to ependymal damage, causing more severe alterations such as disruption of the epithelium and detachment and loss of ependymal cells [21, 49]. The bases of the NA cytotoxicity on ependymal cells are unknown. The NA from *C. perfringens* (Roche, cat. no. 11 585 886 001) contains less than 0.1 % of proteases. It is well established that *C. perfringens* produces a plethora of potent toxins [62], some of which might be included in the contaminating protease fraction. This could represent a putative source of toxicity for ependymal cells. Furthermore, NA is able to enhance the cytotoxicity of some of these toxins [63]. On the other hand, desialylation of glycoprotein receptors or surface proteins involved in cell survival could compromise the viability of ependymal cells.

Cells from the CNS are able to produce complement proteins [25]. However, we discard the possibility that the explants could be the source of complement factors in our experiments because (i) explants are thoroughly washed before the assay and (ii) the length of the experiment is not enough for those factors to accumulate in the culture media.

Ventricular wall explants provide a suitable in vitro model close to the in vivo situation, as the epithelial architecture of the ependymal is preserved. However, the possible influence of the underlying tissue on the ependymal cells cannot be ruled out. Therefore, we proceeded to reproduce the experiment with isolated ependymal cells. The results obtained were similar to those with explants: NA alone reduced the viability of ependymal cells and the addition of serum as a source of complement further reduced viability. This influence of serum was reversed by the addition of a complement inhibitor (C5 inhibitor), confirming the complement specificity of the observed effect and excluding the possibility that other factors present in serum could be responsible.

An alternative experimental approach to test the role of the complement system in the ependymal death induced by NA consisted of the in vivo blockade of C5 activation. C5 is pivotal in the progress of the lytic pathway of complement, and its proteolytic activation initiates the activation cascade that concludes with MAC assembly. Therefore, C5 proteolysis blockade (by using a C5 inhibitor antibody) prevents MAC-mediated cellular lysis [64]. The same inhibitor has been also used systemically in other models of tissue injury, such as hemorrhagic shock [65].

In the present work, C5 inhibitor was administered both systemically and icv to the same animals, with the aim of reinforcing the inhibition at the level of the ventricular system. The C5-inhibited rats that were injected with NA suffered ependymal cell disruption, albeit less than the non-inhibited rats. The damage provoked by NA under C5 inhibition could have several explanations: (i) NA activates the complement system downstream of C5 in the proteolytic cascade, this is unlikely because in vitro experiments show NA-induced activation of complement at the level of C3 and generates iC3b in human serum [33]; (ii) other mechanisms, independent of complement, could account for the damage of ependymal cells; and (iii) the level of inhibition achieved was not enough to avoid the lytic action of complement, the ongoing production of complement factors, both in the blood and in the CSF, and the continuous circulation of the CSF, would allow the escape of a fraction of C5 from inhibition, enough to permit the progress of the lytic pathway at a moderate level. This third explanation seems the most probable because (i) serum from rats treated systemically with anti-C5 had 60–70 % residual hemolytic activity compared to that of the non-inhibited rats (our data; not shown); (ii) published data indicates that the anti-C5 antibody used here is able to reduce the C5 activity of serum more than 50 %, as evaluated by C5b-9-mediated hemolytic assay [39]; and (iii) a minor amount of C5b-9 could be detected by immunohistochemistry on the ependymal surface of the anti-C5-inhibited rats.

As exposed above, the use of functional blocking antibodies against C5 relieve ependymal epithelium disruption in the NA-injected rats. This fact suggests that C5 inhibition is a suitable therapeutic strategy to prevent ependymal loss and ventricular disorganization, which frequently occur after CNS infections that trigger complement activation.

The C6-deficient rats express a natural deletion in the C6 gene [34, 66] that prevents C6 synthesis and formation of MAC and hence the complement-mediated cell lysis [67, 68]. The use of this strain made it possible to evaluate the damage induced by NA on ependymal cells in the absence of MAC formation. Ependymal disruption after NA injection was reduced in the C6-deficient compared to the NA-treated wild-type rats. Since MAC formation is completely precluded in C6 mutant rats (and was not detected by immunohistochemistry), our results lead us to conclude that the lytic pathway of the complement contributes to but is not the only cause for NA-induced ependymal damage.

Various CNS pathologies are caused by microorganisms displaying the enzyme NA on their covers, such as the flu virus [16, 69, 70], the mumps virus [13], and the bacteria *C. perfringens*, which produces meningitis and encephalitis [71]. The rats injected with NA into the cerebral ventricles suffer ependymal cell death [40] and a remarkable neuroinflammatory reaction [9], suggesting an important role of this enzyme in the neurodegenerative events occurring after microbial invasions of the CNS. Bearing in mind the results presented here that NA can directly activate the alternative complement pathway [33], and the demonstrated role of the complement in neurodegenerative processes [25, 54, 72], it may be suggested that NA could be responsible, at least partially, for the neuroinflammatory reaction observed in the icv NA-injected rat model [9] and, more importantly, in the above-mentioned infectious pathologies. Pharmacological targeting of complement molecules could be a strategy for reducing the damage after CNS insults [73–75]. However, it is noteworthy that the complement system is involved in physiological processes both during development [76–78] and in adulthood [79]. For this reason, the design of effective therapeutic strategies based on the specific blockade of complement fragments should take into account the diverse spatiotemporal physiological functions of the different complement fragments [73].

## Conclusions

In summary, in the present work, the detection of MAC complexes on the ependyma of NA-injected rats, together with the demonstration of in vitro activation of C3 by NA, suggests that the complement system contributes to ependymal cell damage and death observed after NA injection into the ventricles. In vitro assays with ventricular wall explants and isolated ependymal cells and in vivo experiments with C6-deficient rats and systemic and central inhibition of C5 activation demonstrate that NA can provoke ependymal damage and cell death even in the absence of complement activation; however, complement activation and MAC formation enhances and exacerbates the cellular damage and epithelial disruption caused by NA. We conclude that complement participates in the ependymal cell death induced by NA but is not the only cause.

## Ethics approval and consent to participate

Animal procedures were performed according to the European Union (86/609/EEC) and Spanish (RD 1201/2005) legislations. Animal care and experimental procedures were approved by the Animal Experimentation Ethics Committee of the University of Málaga (ref. number 2012–0013).

### Consent for publication

Not applicable.

### Availability of data and materials

There is no new software, databases, and application/tool available, apart from the reported in the present article.

## Abbreviations

ABC: avidin-biotin complex
BCA: bicinchoninic acid
C5-inh: C5 complement inhibitor
CM: culture medium
CNS: central nervous system
CSF: cerebrospinal fluid
DAB: diaminobenzidine
icv: intracerebroventricular
MAC: membrane attack complex
MBL: mannose-binding lectin
NA: neuraminidase
PAGE: polyacrylamide gel electrophoresis
PB: phosphate buffer
PBS: phosphate-buffered saline
PNA: peanut (*Arachis hypogaea*) agglutinin
RT: room temperature
